# Evaluation of the corrosion resistance of a Ni-P coating deposited on additive manufacturing steel: A dataset

**DOI:** 10.1016/j.dib.2020.106159

**Published:** 2020-08-08

**Authors:** Dayi Gilberto Agredo Diaz, Arturo Barba Pingarrón, Jhon Jairo Olaya Florez, Jesús Rafael González Parra, Javier Cervantes Cabello, Irma Angarita Moncaleano, Alba Covelo Villar, Miguel Ángel Hernández Gallegos

**Affiliations:** a Universidad Nacional de Colombia-sede Bogotá-Facultad de Ingeniería-Departamento de Ingeniería Mecánica y Mecatrónica-Bogotá, Colombia; b Universidad Nacional Autónoma de México-Facultad de Ingeniería-División de Ingeniería Mecánica e Industrial-Centro de Ingeniería de Superficies y Acabados (CENISA), Ciudad de México, México

**Keywords:** Additive manufacturing, Electroless nickel plating, Electrochemical noise, EIS, Microhardness, X-ray diffraction

## Abstract

This article presents the data set obtained for the research work entitled “Effect of a Ni-P coating on the corrosion resistance of an additive manufacturing carbon steel immersed in a 0.1 M NaCl solution” [1]. Microstructural, mechanical, and electrochemical characterization (using the electrochemical impedance and electrochemical noise spectroscopy technique) is performed on a material obtained by additive manufacturing and the influence of a Ni-P coating on it. The layer sizes and hardness of the substrate are measured, as well as the thickness of the coating and its hardness, values for corrosion resistance, resistance to electrochemical noise and location indices are calculated. The data show an adequate deposition rate for the type of coating, as well as the increase in corrosion resistance when the coating is applied to the steel by additive manufacturing.

**Specifications Table**

**Table:** 

Subject	Surface engineering
Specific subject area	Corrosion
Type of data	Table Image Figure
How data were acquired	SEM images are obtained using a Philips XL20 scanning electron microscope. Layer and coating thicknesses are measured using the free-access Imagej computer tool. The microhardness is obtained in a digital microdurometer model HVS-1000. The structure of the material is characterized by X-ray diffraction and the data is obtained in a PANalytical X´Pert Pro MPD diffractometer. Electrochemical impedance spectroscopy and electrochemical noise data are obtained on a Gill AC potentiostat/galvanostat using Sequencer software.
Data format	Raw Analyzed
Parameters for data collection	Electron microscopy images are obtained with 200 kV secondary electrons with a working distance of 7.4 mm. The microhardness of the additive manufacturing steel layers and the coating is measured by applying a load of 100 g with a time of 10 s. The structure of the coatings was evaluated employing x-ray diffraction at a room temperature of 25°C, a range between 15 and 90° is established, a time per step of 30 s, for a total scanning time of 7 min. In the corrosion study a three-electrode electrochemical cell is used, which is composed of an SCE electrode as reference, a graphite plate as the counter electrode and the sample as a working electrode. The sweep frequency for the electrochemical impedance spectroscopy data was from 10^4^ to 10^−1^, acquiring 10 points/decade, a sine wave is used as a disturbance to the system with amplitude of 10 mV. In electrochemical noise, time series of 1024 points are obtained every 0.5 s. The electrochemical evaluation is carried out in a 0.1 M sodium chloride solution.
Description of data collection	Corrosion data is collected through the use of the Sequencer software. The layer size and coating thickness measurement is collected using the Imagej software. The microhardness data is collected manually. The XRD data is taken by the diffractometer.
Data source location	Institution: Universidad Nacional Autónoma de México, Centro de Ingeniería de Superficies y Acabados (CENISA) City: Ciudad de México Country: México.
Data accessibility	With the article
Related research article	D.G. Agredo Diaz, A. Barba Pingarrón, J.J. Olaya Florez, J.R. González Parra, J. Cervantes Cabello, I. Angarita Moncaleano, A. Covelo Villar, M.Á. Hernández Gallegos, Effect of a Ni-P coating on the corrosion resistance of an additive manufacturing carbon steel immersed in a 0.1 M NaCl solution, Mater. Lett. 275 (2020) 128159. https://doi.org/https://doi.org/10.1016/j.matlet.2020.128159

## Value of the Data

These data provide a look at the corrosion behavior of steel produced by additive manufacturing and the influence of a Ni-P coating applied on the material.
These data provide the ability to predict the influence of chloride ions on additive manufacturing steel and on the applied Ni-P coating and its useful life, being useful for academics and researchers in the area of additive manufacturing, electrochemistry, surface engineering, and corrosion protection.
When evidencing a considerable increase in the corrosion resistance of the coated material, these coatings are proposed as favorable to be applied to this type of substrates under similar working conditions.

## Data Description

**Fig. 1:**
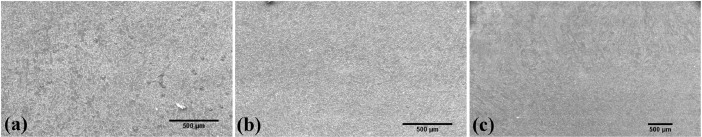
SEM microstructure with secondary electrons of the steel produced by additive manufacturing. (a) Lower zone, (b) Middle zone, (c) Upper zone.

In additive manufacturing steel three important areas were identified, Fig. 1 (scanning electron microscopy images), (a) shows the area of the first 2 layers of the steel by additive manufacturing, (b) shows the intermediate area of the material by additive manufacturing, and (c) shows the upper zone of the steel by additive manufacturing.

The synthesis of the coatings is made using a bath of: Nickel sulfate (*NiSO*_4_) 0.2 M, Sodium hypophosphite (*NaPOH*_2_) 0.25 M, Propionic acid (*C*_3_*H*_6_*O*_2_) 0.1 M, Lactic acid (*C*_3_*H*_6_*O*_3_) 0.25 M, Sodium acetate (*NaC*_2_*H*_3_*O*_2_) 0.3 M, Lead shot 1.13 × 10^−6^ M, the pH of the solution is maintained at 4.5 at a temperature of between 86-88°C, an activation of the test pieces is performed in 15% HCl. The immersion time in the bath was 3.5 h under stirring.

**Fig. 2:**
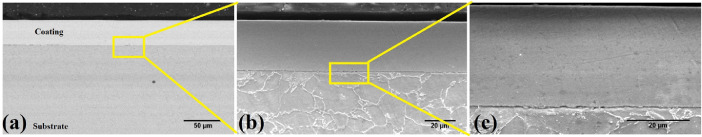
(a) Scanning electron microscope micrograph of backscattered electrons from the substrate and the Ni-P coating, (b) magnification of the highlighted area in a, micrograph by secondary electrons, (c) Magnification of the area marked in b showing a detail of the base metal-coating interface.

**Fig. 3:**
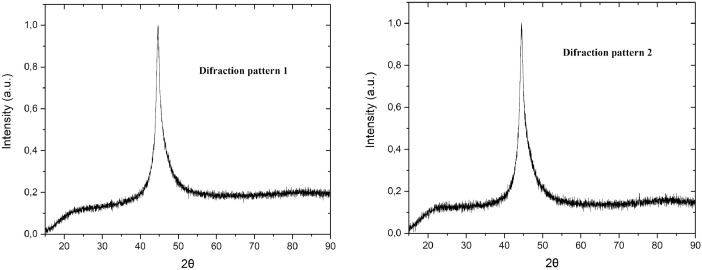
Diffraction patterns for 2 samples coated with electroless nickel plating. Figure reproduced from [1].

Fig. 2 shows an electron microscopy image of the Ni-P layer deposited on the steel, Table 1 shows the data resulting from the measurement of the thickness of the coating, and its distribution in Fig. 3.

**Table 1:** Coating thickness measurement.

Measurement number	Coating thickness (µm)
1	32.06
2	31.85
3	32.70
4	32.49
5	32.06
6	31.81
7	32.49
8	32.28
9	32.22
10	32.36
11	32.50
12	32.50
13	32.22
14	32.50
15	32.78

Table 2 shows the microhardness data of each of the layers of the material produced by additive manufacturing (data resulting from the measurement in the cross-section), and the microhardness data of the coating.

**Table 2:** Microhardness of the additive manufactured steel layers and the Ni-P coating.

	Microhardness (HV)					
Distance from the edge of the layer (µm)	Layer 1	Layer 2	Layer 3	Layer 4	Layer 5	Ni-P coating
500	155.60	156.70	149.60	147.10	151.70	521.20
1000	159.30	147.70	151.10	147.30	162.80	416.50
1500	158.20	146.00	152.30	149.50	148.80	467.90
2000	161.20	145.20	157.10	154.20	163.10	420.70
2500	150.80	156.50	151.30	170.08	155.00	459.00
3000	150.20	147.50	153.30	171.00	161.50	480.56

Table 3 shows the data set of the diffractograms presented in Fig. 3. Fig. 3 shows 2 diffraction patterns obtained for the Ni-P coating of amorphous nature.

**Table 3:** Fig. 3 data set

Difraction pattern 1		Difraction pattern 2	
2Ɵ (°)	Intensity (a.u)	2Ɵ (°)	Intensity (a.u)
15.0000	0.0228	15.0000	0.0248
15.8070	0.0303	15.8185	0.0213
16.5922	0.0303	16.6350	0.0276
17.3871	0.0416	17.4089	0.0526
18.1826	0.0611	18.1959	0.0799
18.9991	0.0601	19.0010	0.0807
19.7783	0.0796	19.8161	0.0980
20.5140	0.0884	20.5780	0.0918
21.3032	0.1077	21.4538	0.1119
22.1232	0.1174	22.2485	0.1163
22.9313	0.1081	22.9886	0.1408
23.7375	0.1181	23.7701	0.1270
24.5137	0.1235	24.4756	0.1386
25.2915	0.1185	25.2608	0.1278
26.0832	0.1313	26.0204	0.1153
26.8478	0.1262	26.8326	0.1278
27.6063	0.1100	27.5818	0.1285
28.3636	0.1319	28.3856	0.1386
29.1435	0.1402	29.1358	0.1276
29.9469	0.1271	29.8811	0.1240
30.7374	0.1419	30.6202	0.1241
31.5140	0.1282	31.3452	0.1171
32.2981	0.1400	32.0888	0.1124
32.9874	0.1464	32.8740	0.1248
33.7687	0.1392	33.6613	0.1325
34.5527	0.1708	34.4507	0.1365
35.3172	0.1519	35.2404	0.1303
36.0685	0.1571	36.0094	0.1376
36.8331	0.1622	36.7690	0.1346
37.6338	0.1639	37.5393	0.1428
38.4307	0.1755	38.3378	0.1460
39.2048	0.1904	39.1190	0.1630
39.9778	0.2027	39.9011	0.1659
40.7321	0.2181	40.6327	0.1765
41.5273	0.2395	41.3830	0.2294
42.2758	0.2541	42.0929	0.2397
43.0835	0.3397	42.8138	0.3276
43.7664	0.4674	43.5670	0.4763
44.3861	0.8381	44.2155	0.8515
44.9083	0.8835	44.7932	0.8073
45.5717	0.5517	45.3865	0.5484
46.3003	0.4510	45.9890	0.4484
47.0219	0.3951	46.6770	0.3752
47.6995	0.3489	47.3521	0.3156
48.4251	0.3106	48.1335	0.2776
49.1769	0.2534	48.8881	0.2295
49.8727	0.2442	49.6219	0.2138
50.5942	0.2275	50.4026	0.2060
51.3638	0.2162	51.1603	0.1806
52.1525	0.2225	51.9043	0.1879
52.8132	0.2044	52.6663	0.1764
53.5926	0.2103	53.4683	0.1578
54.3793	0.1976	54.2186	0.1612
55.1533	0.1921	54.9836	0.1581
55.9468	0.1917	55.7657	0.1479
56.7264	0.1905	56.5392	0.1310
57.4647	0.1914	57.3249	0.1430
58.2636	0.1823	58.0553	0.1544
59.0232	0.1958	58.8460	0.1496
59.7949	0.1669	59.6008	0.1458
60.5238	0.1858	60.3787	0.1378
61.2710	0.1862	61.1145	0.1462
62.0148	0.1827	61.8961	0.1499
62.7540	0.1818	62.6625	0.1473
63.5544	0.1829	63.4540	0.1397
64.3316	0.1864	64.2654	0.1276
65.0819	0.1834	65.0590	0.1414
65.8403	0.1682	65.8042	0.1371
66.5822	0.1825	66.5811	0.1243
67.3155	0.1839	67.3377	0.1491
68.1070	0.1785	68.0820	0.1164
68.8555	0.2024	68.8458	0.1396
69.6171	0.1892	69.5965	0.1401
70.4059	0.1940	70.3481	0.1340
71.1813	0.1941	71.0941	0.1501
71.9316	0.1963	71.8946	0.1238
72.6879	0.1812	72.6120	0.1442
73.4668	0.1931	73.3917	0.1470
74.2347	0.1978	74.1265	0.1479
74.9907	0.1896	74.8925	0.1441
75.7266	0.2006	75.6692	0.1413
76.5005	0.1863	76.4580	0.1645
77.2740	0.1937	77.2201	0.1400
78.0228	0.1810	77.9694	0.1439
78.7839	0.1826	78.8022	0.1548
79.5439	0.2027	79.5596	0.1535
80.3098	0.2020	80.3225	0.1287
81.0769	0.2186	81.0861	0.1532
81.8258	0.1965	81.8747	0.1373
82.6073	0.2051	82.5303	0.1625
83.3202	0.1815	83.2947	0.1568
84.0503	0.1973	84.0311	0.1495
84.7619	0.2086	84.7329	0.1602
85.5028	0.1969	85.4658	0.1696
86.2690	0.1921	86.1902	0.1557
87.0250	0.1908	86.9609	0.1473
87.7567	0.1776	87.7255	0.1528
88.5024	0.1996	88.4629	0.1471
89.2519	0.2003	89.2248	0.1277
90.0000	0.1844	90.0000	0.1366

**Fig. 4:**
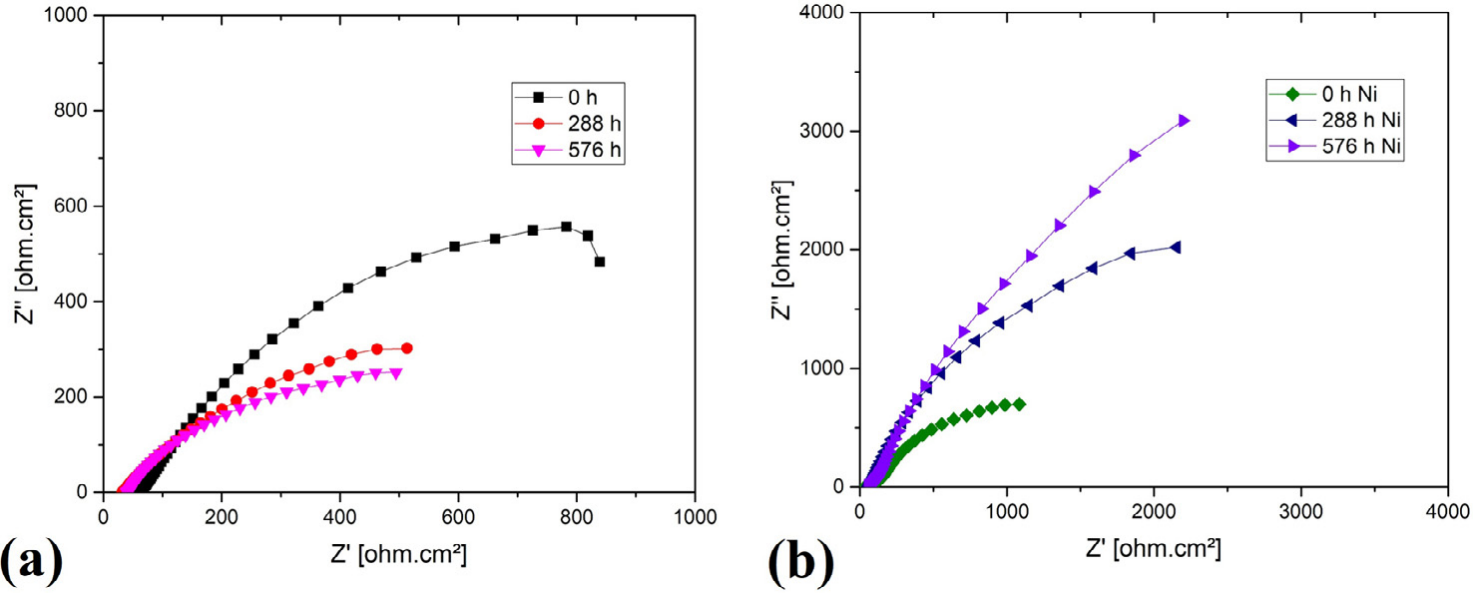
(a) Nyquist diagram for additive manufacturing steel, (a) Nyquist diagram for coated steel. Figure reproduced from [1].

Table 4 shows the data set in Fig. 4 (Z´ is the real impedance, Z´´ is the imaginary impedance). Fig. 4a shows the Nyquist diagram for steel by additive manufacturing, Fig. 4b shows the data for coated steel.

**Table 4:** Fig. 4 data set.

0 h additive manufacturing steel		288 h additive manufacturing steel		576 h additive manufacturing steel		0 h coated steel		288 h coated steel		576 h coated steel	
Z'	Z''	Z'	Z''	Z'	Z''	Z'	Z''	Z'	Z''	Z'	Z''
[Ω.cm²]	[Ω.cm²]	[Ω.cm²]	[Ω.cm²]	[Ω.cm²]	[Ω.cm²]	[Ω.cm²]	[Ω.cm²]	[Ω.cm²]	[Ω.cm²]	[Ω.cm²]	[Ω.cm²]
56.5950	0.2142	31.6840	0.0357	39.4570	0.1804	37.2010	3.1441	60.4330	3.9140	54.9420	8.0075
57.0340	0.6888	31.8820	0.4513	39.7240	0.6391	37.4810	3.2436	60.8500	3.5201	56.3370	7.7079
57.3870	1.2404	31.9910	0.6193	39.8800	0.8385	37.7450	3.3805	61.2400	3.0543	57.4220	7.2753
57.6550	1.5640	31.9760	0.5090	39.8770	0.6975	37.9930	3.5241	61.3670	2.0232	58.1120	6.0185
58.2890	1.8650	32.0580	0.5121	39.9510	0.8985	38.3530	3.7790	61.5660	1.1085	58.6420	5.1779
58.6770	1.8270	32.0760	0.5827	39.9470	0.9734	38.6670	4.0526	61.6270	0.4384	59.1470	4.4886
58.9060	1.8443	32.0380	0.7145	40.0330	1.0756	38.9750	4.3710	61.7480	0.4483	59.6940	4.7793
59.0190	2.3242	32.0530	0.8326	40.0640	1.1393	39.3770	5.1530	61.9040	0.8871	60.3020	5.0986
59.2850	2.7993	32.1230	1.0066	40.1970	1.3074	39.8030	5.8931	61.9760	1.3273	60.9810	5.5717
59.5710	3.1939	32.3030	1.1651	40.2050	1.5567	40.2820	6.7668	62.1990	1.8378	61.5290	5.8527
59.8230	3.4592	32.3240	1.4621	40.2570	1.7678	40.7590	7.5229	62.3210	2.1811	62.0670	6.1428
60.1130	3.7358	32.3630	1.6581	40.3190	2.0610	41.3020	8.5060	62.3740	2.5594	62.5560	6.6446
60.5560	4.1440	32.4430	1.9760	40.5510	2.3017	42.0840	9.2413	62.4290	2.8908	63.3130	7.2353
60.9060	4.4472	32.7130	2.1104	40.8500	2.4421	42.8620	10.1350	62.5650	3.1126	64.0480	7.6523
61.3220	4.8060	32.9650	2.3624	41.1580	2.5730	43.8750	11.1970	63.0880	3.3841	64.8690	8.0336
62.2320	5.0902	33.7390	2.4508	41.9550	2.6261	44.8970	12.8570	63.5570	3.7678	65.6720	8.6560
62.9550	5.5946	34.4900	2.7520	42.5360	2.7825	45.9370	14.6220	63.6280	4.2786	66.1240	9.5456
63.8410	6.2837	35.3060	3.0993	43.3030	3.0679	47.1910	16.7220	63.6000	5.1004	66.8040	10.6420
64.2320	7.0516	35.6100	3.6383	43.4540	3.6338	48.6210	18.8520	63.8040	5.9528	67.5930	12.0210
65.2080	7.7969	35.8490	4.0982	43.7860	4.1344	50.3280	20.9100	64.6840	6.8118	68.9340	13.2340
66.2400	8.7970	36.1350	4.7170	44.0780	4.6711	52.7770	23.5010	65.5760	7.8196	70.2570	14.7620
67.3910	10.0230	36.4550	5.3978	44.5330	5.2129	55.5720	26.3390	66.3190	8.9810	71.6670	16.5050
68.5300	11.5470	36.8950	6.3676	44.9660	6.0865	59.3250	30.0700	66.9550	10.6450	73.1560	19.0490
69.4960	12.9490	37.3980	7.3580	45.4440	6.9481	63.2180	33.1730	67.5310	12.2280	74.6770	21.6010
70.6300	14.6050	38.1530	8.5250	46.0870	7.9477	68.1540	36.5260	68.3720	14.3510	76.4500	24.5900
71.5270	16.1230	38.7680	9.5823	46.6330	8.8473	72.5710	39.1670	69.0940	16.3790	78.0630	27.4190
73.0730	18.5330	39.7720	11.1280	47.3980	10.2500	78.9230	42.2280	70.3020	19.6640	80.7270	31.6780
74.5130	20.8530	40.6050	12.6860	48.0630	11.7530	84.6430	44.7140	71.3730	22.8930	83.4040	35.8640
76.3900	23.7750	41.7860	14.5900	48.9220	13.6170	91.4960	46.9180	72.6800	26.8690	86.6690	41.1550
77.7980	26.1560	42.7820	16.0980	49.6080	15.2380	96.3650	48.2190	73.9160	30.0160	89.4010	45.4900
79.7940	29.5550	44.1910	18.2040	50.6710	17.3870	102.3000	49.5530	75.6620	34.6140	93.1490	51.5260
81.8680	33.4130	45.7780	20.5300	51.9440	19.8900	108.8400	51.4760	77.6520	40.1020	98.0860	58.6740
84.5590	38.1700	47.7480	23.4170	53.5150	22.9620	115.6600	53.6210	79.9790	46.9750	103.7600	67.3680
87.1310	42.9760	49.6830	26.1380	55.1080	26.0300	121.6500	55.8660	82.4140	54.0630	109.3800	76.1390
90.4620	48.8000	52.0910	29.4330	57.1310	29.9660	126.7400	58.1680	86.2550	63.3660	114.8800	86.3050
94.0510	55.5640	54.7650	32.9940	59.7020	34.3570	131.8300	61.3360	90.5780	74.3610	120.6800	97.6950
98.3130	63.3100	58.1300	37.1910	62.9260	39.5570	136.9800	65.2750	95.7580	87.5410	127.0900	110.9700
102.8400	72.0080	61.7550	41.7210	66.5170	44.9350	142.1100	70.0560	100.6900	102.0200	134.1700	126.2800
108.3700	81.7890	65.9090	46.6840	70.7170	50.9670	147.3200	75.9580	106.5300	119.0300	141.9100	144.5400
114.4900	92.9170	70.3430	52.1120	75.5260	57.4670	152.8200	83.4030	113.0700	138.6200	150.2500	165.9900
121.7000	105.5400	75.3790	57.9730	81.2060	64.5170	158.9000	92.8640	120.8800	161.5100	159.3100	191.1900
129.9100	119.7300	80.9100	64.4060	87.6830	72.1630	165.5300	104.3500	130.2000	187.8600	169.6300	220.9300
139.7500	136.1000	87.2670	71.7010	95.0410	80.6450	173.0600	118.3500	140.9500	218.6400	181.8500	256.6500
151.3200	154.4000	94.7340	79.6930	103.9700	89.8950	182.0200	135.0500	155.2700	255.2200	196.8000	298.9400
165.9200	176.4400	102.7900	88.5680	114.0900	99.3830	192.2200	154.3400	171.9900	297.0600	213.8800	347.1500
183.2000	200.8900	112.1100	97.8960	125.5000	109.3300	204.7400	176.8200	192.6900	345.9200	235.1900	405.0200
204.2200	228.7900	122.6200	108.5200	138.6600	119.7300	220.3600	203.1500	217.5300	402.1400	261.7600	471.9600
228.1600	258.5500	135.1800	120.1200	153.5800	130.9900	240.2600	234.0900	248.6300	469.8500	296.6600	553.1600
255.2600	289.2300	148.7000	132.2200	170.1300	142.0200	264.8000	268.2500	286.0400	544.9900	336.5000	640.8100
285.7900	321.1000	163.8300	145.3300	187.6400	152.9100	293.6700	305.3300	332.1500	629.8200	384.2000	740.9500
321.8300	354.4600	180.7700	159.1100	207.1800	163.7600	329.4300	345.4900	389.8900	724.6000	439.9700	853.2600
363.5600	390.8900	200.3200	174.4900	230.6800	176.4500	372.1600	389.7100	461.0600	835.7700	510.9000	987.4200
413.8900	428.3500	224.8000	192.1700	256.5500	189.3100	425.0800	436.6700	550.5200	959.9300	597.2900	1,140.4000
469.1500	462.8200	251.6800	210.3600	283.0600	201.1400	487.0500	483.6300	660.2900	1,094.5000	701.6600	1,310.2000
528.8900	492.4600	282.1900	228.8000	309.7600	210.9100	557.1300	528.6300	787.7300	1,231.8000	827.6700	1,503.0000
594.0200	515.8300	313.2300	244.6800	338.0400	218.5900	638.3700	569.1500	952.9400	1,382.9000	977.5200	1,713.9000
662.3800	531.2800	347.6100	258.8500	369.2000	226.3500	725.9500	603.2500	1,146.8000	1,528.6000	1,159.2000	1,946.6000
725.9600	548.8900	382.1700	275.1800	399.2500	235.6900	812.7000	639.5600	1,360.0000	1,695.4000	1,353.2000	2,203.3000
782.7800	556.8600	419.5600	289.0500	429.4900	244.9700	898.6800	668.7700	1,585.8000	1,843.3000	1,587.1000	2,490.3000
819.0300	537.9200	462.7400	300.1900	460.3600	251.5000	986.6800	693.0000	1,842.4000	1,969.2000	1,860.6000	2,795.4000
839.3100	482.8100	513.4800	301.8900	494.5300	252.1100	1,084.5000	698.3400	2,152.8000	2,020.7000	2,196.6000	3,089.3000

**Fig. 5:**
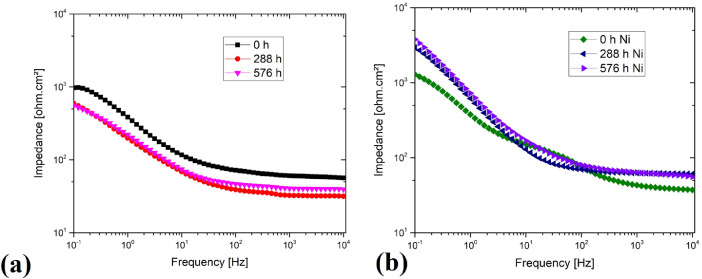
(a) Impedance bode diagram for additive manufacturing steel, (a) Impedance bode diagram for coated steel. Figure reproduced from [1].

Table 5 shows the data for the impedance bode diagram in Fig. 5. Fig. 5a shows the data in the impedance boundary diagram for the uncoated steel and Fig. 5b for the coated material.

**Table 5:** Fig. 5 data set.

	0 h additive manufacturing steel	288 h additive manufacturing steel	576 h additive manufacturing steel	0 h coated steel	288 h coated steel	576 h coated steel
Frequency [Hz]	Impedance [Ω.cm²]					
10,000.000	56.595	31.684	39.458	37.334	60.559	55.522
8,254.000	57.039	31.885	39.729	37.622	60.952	56.862
6,812.900	57.400	31.997	39.889	37.896	61.317	57.882
5,623.400	57.677	31.980	39.883	38.156	61.400	58.422
4,641.500	58.319	32.062	39.961	38.539	61.576	58.870
3,831.100	58.705	32.081	39.959	38.879	61.628	59.317
3,162.200	58.935	32.046	40.048	39.220	61.750	59.885
2,610.100	59.065	32.064	40.080	39.712	61.910	60.518
2,154.400	59.351	32.139	40.219	40.237	61.990	61.235
1,778.200	59.657	32.324	40.235	40.846	62.226	61.807
1,467.700	59.923	32.357	40.296	41.448	62.359	62.370
1,211.500	60.229	32.406	40.371	42.169	62.427	62.908
1,000.000	60.698	32.504	40.617	43.087	62.495	63.725
825.400	61.068	32.781	40.923	44.044	62.643	64.504
681.290	61.510	33.050	41.239	45.282	63.179	65.365
562.340	62.440	33.828	42.037	46.702	63.669	66.240
464.150	63.203	34.600	42.626	48.208	63.771	66.809
383.110	64.149	35.442	43.411	50.067	63.804	67.646
316.220	64.618	35.795	43.606	52.147	64.081	68.654
261.010	65.673	36.082	43.981	54.499	65.042	70.193
215.440	66.821	36.442	44.325	57.773	66.040	71.791
177.820	68.133	36.852	44.837	61.498	66.925	73.544
146.770	69.496	37.440	45.376	66.511	67.796	75.596
121.150	70.692	38.115	45.972	71.393	68.630	77.738
100.000	72.125	39.094	46.767	77.325	69.862	80.308
82.540	73.322	39.935	47.465	82.466	71.009	82.739
68.129	75.386	41.300	48.494	89.510	73.000	86.720
56.234	77.376	42.541	49.479	95.728	74.955	90.788
46.415	80.004	44.260	50.782	102.820	77.487	95.944
38.311	82.077	45.710	51.896	107.750	79.778	100.310
31.622	85.092	47.794	53.571	113.670	83.204	106.450
26.101	88.424	50.171	55.622	120.400	87.396	114.290
21.544	92.775	53.181	58.234	127.480	92.754	123.710
17.782	97.153	56.139	60.946	133.870	98.564	133.270
14.677	102.780	59.831	64.513	139.450	107.020	143.690
12.115	109.230	63.936	68.883	145.400	117.190	155.270
10.000	116.930	69.009	74.327	151.740	129.740	168.720
8.254	125.540	74.528	80.273	158.440	143.340	184.250
6.813	135.770	80.768	87.170	165.750	159.740	202.560
5.623	147.450	87.543	94.904	174.100	178.890	223.890
4.642	161.100	95.094	103.710	184.040	201.740	248.860
3.831	176.680	103.410	113.560	195.680	228.570	278.550
3.162	195.070	112.940	124.640	209.660	260.140	314.540
2.610	216.190	123.790	137.450	226.650	298.750	357.910
2.154	242.200	135.690	151.310	246.520	343.260	407.750
1.778	271.880	148.840	166.450	270.530	395.960	468.350
1.468	306.680	163.750	183.200	299.720	457.200	539.690
1.212	344.820	180.840	201.860	335.450	531.580	627.690
1.000	385.770	198.980	221.620	376.940	615.490	723.790
0.825	429.870	219.000	242.060	423.640	712.040	834.640
0.681	478.770	240.820	264.090	477.370	822.830	960.010
0.562	533.830	265.660	290.430	538.870	954.510	1,111.700
0.464	595.650	295.740	318.830	609.410	1,106.500	1,287.400
0.383	659.020	328.020	347.250	686.380	1,278.300	1,486.200
0.316	722.670	363.290	374.740	768.020	1,462.100	1,715.800
0.261	786.730	397.480	402.560	855.250	1,679.400	1,973.100
0.215	849.130	433.400	433.060	943.890	1,910.900	2,265.600
0.178	910.110	470.940	463.630	1,034.100	2,173.500	2,585.700
0.147	960.650	509.490	494.440	1,120.200	2,431.600	2,953.100
0.121	979.890	551.590	524.580	1,205.700	2,696.700	3,358.000
0.100	968.270	595.650	555.090	1,289.900	2,952.600	3,790.700

**Fig. 6:**
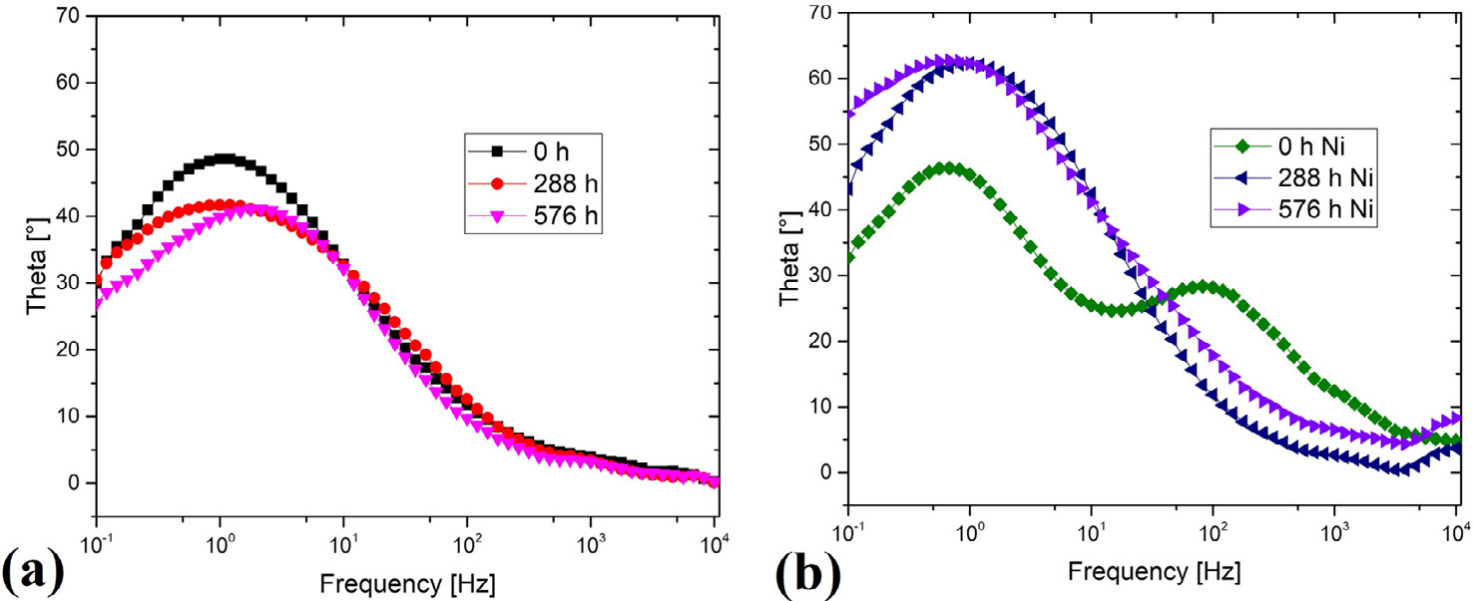
(a) Phase angle bode diagram for additive manufacturing steel, (a) Phase angle bode diagram for coated steel. Figure reproduced from [1].

Table 6 shows the data for the phase angle bode diagram of Fig. 6. Fig. 6a shows the data for the phase angle bode diagram for additive manufacturing steel, Fig. 6b shows the data for the coated material.

Table 7 shows the noise resistance data and the location index calculation for steel by additive manufacturing and Ni-P coating [2].

## Experimental Design, Materials, and Methods

SEM images are obtained in a Philips XL 20 electron microscope at an acceleration voltage of 200 kV and a WD of 7.4 mm, the metallographic preparation is carried out using conventional polishing techniques based on the ASTM E3 [3], the attack Chemical used to reveal the microstructure was 2% Nital. Coating thickness is measured from SEM images in free software ImageJ [4].

The microhardness data is obtained in a digital microdurometer model HVS 1000 following the guidelines of the ASTM E384 standard [5] using a 100 g load at 10 s hold with a diamond tip vickers indenter.

Fig. 3 shows the diffractograms for 2 samples of the electroless nickel plating steel, these are obtained in a PANalytical X'PERT PRO MPD X-ray diffractometer in a range of 15 ° to 90 °, with time per step of 30 s, and a total scan time of 7 min, this test is applied at an ambient temperature of 25°C.

**Table 6:** Fig. 6 data set.

	0 h additive manufacturing steel	288 h additive manufacturing steel	576 h additive manufacturing steel	0 h coated steel	288 h coated steel	576 h coated steel
Frequency [Hz]	Theta [°]					
10,000.000	0.21684	0.06459	0.26199	4.831	3.7057	8.2923
8,254.000	0.69196	0.81093	0.92175	4.946	3.3108	7.7908
6,812.900	1.2382	1.109	1.2045	5.1179	2.8552	7.2208
5,623.400	1.5539	0.91186	1.002	5.2995	1.8883	5.913
4,641.500	1.8326	0.91519	1.2883	5.6273	1.0315	5.046
3,831.100	1.7834	1.0407	1.3958	5.9834	0.40762	4.3398
3,162.200	1.7933	1.2775	1.5391	6.3989	0.41593	4.5775
2,610.100	2.2552	1.488	1.6289	7.4557	0.82103	4.833
2,154.400	2.7034	1.7949	1.8629	8.422	1.2269	5.2205
1,778.200	3.069	2.0657	2.2175	9.5361	1.6924	5.4337
1,467.700	3.3094	2.5899	2.5144	10.457	2.0044	5.6523
1,211.500	3.5562	2.9329	2.9264	11.637	2.3497	6.0632
1,000.000	3.9148	3.4854	3.2487	12.385	2.6512	6.5195
825.400	4.1762	3.6913	3.4212	13.304	2.8481	6.8133
681.290	4.4814	4.0992	3.5772	14.317	3.0704	7.0599
562.340	4.6761	4.1547	3.5817	15.98	3.3927	7.5087
464.150	5.0784	4.562	3.7427	17.657	3.8471	8.2145
383.110	5.6215	5.0169	4.0526	19.511	4.5851	9.0514
316.220	6.2651	5.8337	4.7802	21.193	5.3302	10.085
261.010	6.8185	6.5218	5.3941	22.562	6.0116	10.868
215.440	7.565	7.4374	6.0494	24.003	6.8003	11.866
177.820	8.4603	8.4226	6.6765	25.359	7.7123	12.969
146.770	9.5651	9.7922	7.7087	26.88	9.0345	14.595
121.150	10.555	11.13	8.6929	27.688	10.264	16.133
100.000	11.683	12.595	9.7847	28.189	11.854	17.83
82.540	12.703	13.883	10.742	28.356	13.336	19.353
68.129	14.231	15.631	12.203	28.15	15.627	21.425
56.234	15.635	17.35	13.741	27.846	17.784	23.268
46.415	17.288	19.247	15.554	27.149	20.289	25.401
38.311	18.583	20.621	17.076	26.582	22.101	26.968
31.622	20.324	22.39	18.939	25.844	24.584	28.95
26.101	22.202	24.154	20.953	25.311	27.314	30.888
21.544	24.294	26.125	23.223	24.873	30.428	32.993
17.782	26.254	27.749	25.284	24.665	33.265	34.841
14.677	28.345	29.468	27.678	24.652	36.303	36.915
12.115	30.574	31.068	29.919	24.95	39.385	38.99
10.000	32.78	32.611	32.155	25.478	42.434	41.127
8.254	34.998	34.043	34.041	26.242	45.377	43.263
6.813	37.042	35.311	35.781	27.275	48.172	45.527
5.623	39.062	36.532	37.268	28.623	50.797	47.85
4.642	40.933	37.564	38.467	30.303	53.188	50.197
3.831	42.665	38.521	39.455	32.228	55.276	52.483
3.162	44.244	39.408	40.316	34.368	57.191	54.681
2.610	45.578	40.072	40.846	36.574	58.685	56.643
2.154	46.76	40.747	41.058	38.763	59.93	58.363
1.778	47.638	41.127	41.063	40.816	60.881	59.857
1.468	48.248	41.508	40.811	42.673	61.59	60.987
1.212	48.574	41.626	40.46	44.255	62.114	61.796
1.000	48.57	41.643	39.855	45.371	62.308	62.296
0.825	48.33	41.577	39.177	46.116	62.195	62.593
0.681	47.763	41.355	38.324	46.364	61.717	62.724
0.562	47.075	41.058	37.413	46.32	61.117	62.643
0.464	45.984	40.526	36.424	45.771	60.166	62.359
0.383	44.611	39.89	35.398	44.798	58.901	61.83
0.316	42.958	39.035	34.251	43.497	57.403	61.161
0.261	40.97	37.996	32.889	41.72	55.431	60.303
0.215	38.733	36.675	31.512	39.726	53.121	59.227
0.178	37.093	35.756	30.555	38.202	51.265	58.444
0.147	35.428	34.564	29.7	36.656	49.295	57.491
0.121	33.296	32.973	28.648	35.083	46.906	56.353
0.100	29.91	30.453	27.013	32.778	43.187	54.586

The monitoring of the corrosion state is carried out using the non-destructive techniques of electrochemical impedance and electrochemical noise spectroscopy, for this purpose a Gill AC potentiostat/galvanostat is used. A three-electrode electrochemical cell is implemented, composed by a SCE electrode as reference, a graphite plate as counter electrode and the specimen as working electrode. The working electrolyte was 0.1 M NaCl (low-medium aggressive corrosion) [6]. The sweep frequency for the electrochemical impedance spectroscopy data was from 10^4^ to 10^−1^, acquiring 10 points/decade, a sine wave is used as a disturbance to the system with amplitude of 10 mV. In electrochemical noise, time series of 1024 points are obtained every 0.5 s. The DC trend of the time series was eliminated using linear methods reported by Lentka and Smulko in [7].

**Table 7:** Noise resistance and location index data for uncoated and coated additive manufacturing steel.

	Niose Resistance (kΩ-cm²)		Localization index	
Time (h)	Additive manufacturing steel	Coated steel	Additive manufacturing steel	Coated steel
0	1.4944	10.0374	0.9445	0.5077
168	0.3172	12.7806	0.8261	0.9997
240	0.1798	19.4271	0.6448	0.9333
360	0.1441	12.8104	0.9803	0.9383
480	0.2197	25.6477	0.9650	0.3289

## Declaration of Competing Interest

The authors declare that they have no known competing financial interests or personal relationships which have, or could be perceived to have, influenced the work reported in this article.
